# Robotic Thoracic Surgery: Current Landscape and Future Directions

**DOI:** 10.1093/icvts/ivag009

**Published:** 2026-01-08

**Authors:** Akshay J Patel, Kazuhiro Yasufuku, Andrea Bille

**Affiliations:** Division of Thoracic Surgery, Toronto General Hospital, Toronto, ON M5G 2C4, Canada; Institute of Immunology and Immunotherapy, University of Birmingham, Birmingham B15 2TT, United Kingdom; Division of Thoracic Surgery, Toronto General Hospital, Toronto, ON M5G 2C4, Canada; Department of Thoracic Surgery, Guy’s Hospital, Guy’s and St. Thomas’ NHS Foundation Trust, London SE1 9RT, United Kingdom; School of Cancer & Pharmaceutical Sciences, King’s College London, London, WC2R 2LS, United Kingdom

**Keywords:** robotic-assisted thoracic surgery (RATS), single-port robotic platforms (SP), surgical training and education

## Abstract

**Objectives:**

Robotic-assisted thoracic surgery (RATS) has transitioned from an emerging adjunct to an established component of minimally invasive thoracic practice. Advances in multi-platform systems, single-port (SP) technology, and artificial intelligence (AI)-driven analytics are shaping the next phase of surgical precision, ergonomics, and patient-specific approaches.

**Methods:**

We synthesized evidence from randomized controlled trials (RCTs), observational series, national training initiatives, and recent technological innovations to assess the current status, potential benefits, limitations, and future trajectory of RATS. Data sources included RVlob, ROMAN, and RAVAL-4 RCTs; the SORTS UK national training survey; our own thymic resection comparative series; and SP platform feasibility studies.

**Results:**

RCT data demonstrate oncological equivalence between RATS and video-assisted thoracoscopic surgery (VATS), with some evidence for improved lymph node yield. National survey findings underscore the need for structured training pathways to meet evolving technical demands. Early SP platform experiences indicate feasibility and safety in high-volume centres, but widespread adoption is constrained by limited availability, instrumentation, and independent validation. AI-enhanced surgical planning and intraoperative navigation are promising adjuncts, though their clinical impact remains to be defined. Cost, access inequities, and reproducibility outside expert centres continue to pose challenges.

**Conclusions:**

The trajectory of RATS is towards the integration of precision oncology, less invasive access strategies, and data-driven surgical intelligence. Realizing this vision will require rigorous comparative studies, equitable access, and incorporation into structured training to ensure safe, reproducible, and patient-centred adoption worldwide.

## Graphical abstract

**Figure:**
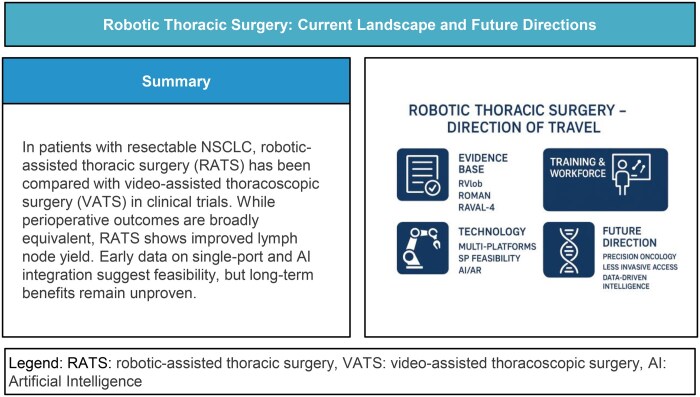

## INTRODUCTION

Robotic-assisted thoracic surgery (RATS) has evolved from a niche innovation to a mainstream surgical modality, driven by advances in instrumentation, imaging, and ergonomics that extend the principles of minimally invasive surgery (MIS). Since its first applications in lung and mediastinal surgery in the early 2000s, RATS has been embraced by high-volume centres worldwide for its potential to combine the minimally invasive benefits of video-assisted thoracic surgery (VATS) with superior dexterity and 3-dimensional visualization.^1–3^ Data from some of the earliest series reinforced the safety and reproducibility of the RATS approach and indeed RATS is currently established as a mainstream surgical modality with supporting data.^4^ From the 2025 latest STS Database report, among database participants, robotic surgery was the most frequent approach to lobectomy and segmentectomy in 2023.^4^ Robotic surgery was also used in a large proportion of oesophagectomies that same year, and data from early groups demonstrate its firm point of utility in the setting of lung resections.^1^^,^^5^

The current trajectory of robotic thoracic surgery is weighted on 3 main elements: (1) technological innovation, with the advent of numerous multi-platform and single-port (SP) systems challenging the traditional dominance of the da Vinci platform; (2) an evolving evidence base, with comparative and prospective studies including the RAVAL, RV-Lob and ROMAN trials^6–8^ and the upcoming RAVAL-4 trial led by McMaster University, aimed at defining the true clinical and oncological benefits of RATS^9^; and (3) training and workforce development, exemplified by initiatives such as SORTS UK, a national survey assessing the integration of robotics into the UK cardiothoracic (CTh) training curriculum and training integration assessments that have been carried out by the Society of Thoracic Surgeons.^10^

This review aims to provide a state-of-the-art overview of RATS, exploring its current clinical landscape, expanding indications, emerging technologies, and future directions. By examining global trends, new platforms, precision surgery concepts, and economic and training considerations, we aim to delineate the “direction of travel” for robotic thoracic surgery over the next decade.

## METHODS

This article was conducted as a narrative review with the aim of synthesizing key developments in robotic thoracic surgery, including randomized evidence, emerging technologies, training frameworks, and future directions. A structured but non-systematic search strategy was used to identify relevant publications.

### Search strategy and sources

A literature search was performed in PubMed, MEDLINE, Embase, and Scopus, complemented by targeted searches of *ICVTS*, *EJCTS*, *JTCVS*, and the *Annals of Thoracic Surgery* to ensure inclusion of high-impact thoracic surgery studies. Additional sources included major conference abstracts (AATS, ESTS, STS, and SCTS), regulatory reports, and manufacturer-published technical white papers where appropriate (eg, new robotic platforms).

### Time frame

Evidence published between January 2010 and July 2025 was considered, capturing the modern era of clinical adoption, platform diversification, and emerging AI-integrated workflows. Studies were included if they reported clinical, technical, or economic outcomes related to robotic thoracic surgery, including lung, mediastinal, or oesophageal procedures; presented randomized trials, prospective/retrospective cohorts, large database analyses, technology feasibility studies, or training/education frameworks; provided data pertaining to multi-arm systems, single-port platforms, or next-generation technologies (AI, AR, automation, performance metrics). Studies were excluded if they reported purely experimental laboratory data without clinical correlation; described paediatric or non-thoracic robotic procedures; lacked extractable outcome data or were superseded by updated analyses from the same cohort.

### Study selection and synthesis

Titles and abstracts were screened for relevance by the lead author, with full-text review performed for all potentially eligible studies. Emphasis was placed on randomized evidence (RVlob, ROMAN, RAVAL-4), large multi-institutional series, and studies offering insight into platform-specific performance, training implications, and technological trajectories. Data were synthesized narratively, structured around thematic domains: evidence base, single-port platforms, global robotic ecosystem, training and credentialing, and emerging digital technologies. **Table 1** provides a synthesis of the manuscripts core sections.

**Table 1.** A Focused Summary of Key Points From the Commentary

Domain	Key points
Evidence base (RCTs)	• RATS is oncologically non-inferior to VATS in RVlob, with comparable perioperative outcomes and higher lymph node yield. • ROMAN confirms similar safety but improved nodal assessment with RATS. • RAVAL-4 shows early cost-effectiveness and comparable patient-reported outcomes.
Single-Port (SP) platforms	• Early feasibility shown by Zervos *et al.* (19 SP lobectomies; 0 conversions; 100% R0). • Over 100 SP cases reported by Lee *et al.*, showing acceptable safety and a learning plateau at ∼20-25 cases. • Adoption remains limited to specialist centres; wider reproducibility not yet established.
Technology and innovation	• AI-assisted augmented reality, automated performance metrics, and 3D reconstruction are emerging adjuncts that may enhance planning and intraoperative navigation. • Clinical impact remains early-phase and requires independent validation.
Training and workforce	• SORTS UK demonstrates low national exposure and highlights the need for formal curricula, simulation, and competency-based credentialing. • Learning curves vary by platform and procedure, reinforcing the need for structured training pathways.
Global platforms and access	• Multi-platform expansion (Hugo RAS, Avatera, Hinotori) may improve competition and access. • Cost and infrastructure remain major determinants of adoption worldwide.
Direction of travel	• Integration of precision oncology, less invasive access strategies (SP), and data-driven surgical intelligence. • Emphasis on equitable implementation, robust comparative evidence, and reproducible training systems.

## CURRENT CLINICAL LANDSCAPE

RATS is now well established for anatomical lung resections (lobectomy and segmentectomy), mediastinal procedures, and increasingly complex oncologic operations such as bronchoplastic and chest wall resections.^3^^,^^11–13^ The United States and South Korea remain leaders in case volumes, while European adoption is accelerating, with significant progress in countries such as the UK driven by centralized robotic programs and institutional investments^14–16^ and Japan, driven by increased reimbursement and funding for robotic procedures.^17^

Major drivers of this expansion include the ergonomic and technical advantages of robotic systems, which allow for precise dissection, improved visualization of hilar structures, and stable instrument control and increased availability of dedicated resources and infrastructure which allows for robust investment in minimally invasive surgical practice. While long-term oncological outcomes remain broadly equivalent to VATS for early-stage lung cancer, RATS is associated with superior lymph node upstaging rates and comparable or reduced postoperative morbidity.^6–8^^,^^18^^,^^19^

Recent data from the European Society of Thoracic Surgery (ESTS) data registry has highlighted significant advantages of the robotic platform in the setting of mediastinal surgery, in terms of enhanced visualization, and higher odds of complete thymic resection (R0) whilst still maintaining equivocal early and late post-operative outcomes when compared to traditional minimally invasive techniques.^18^ Given the anatomical constraints in the mediastinum, the versatility and precision of the RATS technique is important.

Numerous robotic systems now exist, with the landscape diversifying beyond the da Vinci system. Systems like the Medtronic Hugo (not yet formally approved for thoracic surgery in Europe) and CMR Versius (a comparatively smaller platform to the Hugo and da Vinci systems) have gained traction in Europe and Asia.^20^ These systems offer modularity, smaller footprints, and cost efficiencies that could broaden institutional adoption. Although clinical outcome data remain limited, early series suggest comparable safety and feasibility to established platforms.^20^ Systems like the M7, Microsurge, da Vinci dV5, ALF-X, Hugo, Versius, and Bitrack are pushing the envelope further with the integration of haptic feedback, a feature or lack thereof in the earlier systems was thought to be the Achille’s heel of robotic surgery. Furthermore, the addition of robotic guidance systems for interventional bronchoscopy, Monarch platform by Aurius health in 2018 and Ion Endoluminal System by Intuitive Surgical in 2019, has the potential to increase localization percentage close to 90% and increase the diagnostic yields and precisely puncture peripheral nodules.^21^ Adjudicated diagnostic yield for the Monarch platform from the TARGET trial was 61.6% and sensitivity to detect malignancy was 78.8%.^22^ Single-port (SP) robotic systems are being heralded as the next frontier in MIS^23^ and as we shall discuss later, early results suggest that SP robotics may combine the benefits of uniportal VATS with the precision of robotics, though long-term outcomes remain to be established.

## EXPANDING INDICATIONS AND EVIDENCE BASE

Robotic thoracic surgery is increasingly applied to complex resections once deemed unsuitable for minimally invasive approaches. These include sleeve lobectomies, carinal reconstructions, and extended chest wall resections, where robotic dexterity can facilitate precise suturing and dissection in confined spaces.^13^

The landmark CALGB 140503 and JCOG0802 trials demonstrated that sublobar resections (particularly anatomical segmentectomy) offer non-inferior survival compared to lobectomy for small (≤2 cm) peripheral non-small cell lung cancers.^24^^,^^25^ This has accelerated interest in robotic segmentectomy, which is well-suited to complex segmental anatomy due to its enhanced visualization and articulation. Robotic-assisted segmentectomy is now increasingly regarded as a preferred minimally invasive option for early-stage lung cancer in high-volume centres.

Three recent randomized controlled trials (RCTs) have evaluated the safety and effectiveness of RATS compared to VATS or open surgery in patients undergoing pulmonary lobectomy for early-stage NSCLC. The RVlob trial^7^^,^^8^ (NCT03134534) conducted at Ruijin Hospital, Shanghai randomized 320 patients with resectable NSCLC to undergo robotic-assisted lobectomy (RAL, *n* = 157) or video-assisted lobectomy (VAL, *n* = 163). Perioperative outcomes, including hospital stay (*P* = .76) and complication rates (*P* = .45), were similar between groups. However, RAL was associated with a significantly higher chest drain output (830 ml vs 685 ml; *P* = .007), greater lymph node yield [median 11 vs 10 nodes; *P* = .02], more N1 nodes sampled (6 vs 5; *P* = .005), and more nodal stations examined (6 vs 5; *P* < .001), albeit at a higher median cost of hospitalization (USD $12 821 vs $8009; *P* < .001). After a median follow-up of 58.0 months, the 3-year overall survival was 94.6% for RAL and 91.5% for VAL (HR 0.65; 95% CI 0.33-1.28; *P* = .21), meeting the predefined criteria for non-inferiority (absolute difference 2.96%; one-sided 90% CI −1.39% to ∞; *P* = .0029). The 3-year disease-free survival was also comparable (88.7% vs 85.4%; HR 0.87; 95% CI 0.50-1.52; *P* = .62). These findings confirm that RAL is non-inferior to VAL in terms of both perioperative safety and long-term oncologic efficacy and may offer advantages in lymphadenectomy quality.

The ROMAN trial^26^ was the first prospective international randomized controlled trial directly comparing RATS to VATS for NSCLC; patients with clinical stage T1-T2, N0-N1 disease were randomized to undergo resection via RATS (*n* = 38) or VATS (*n* = 39). The primary outcome was the incidence of adverse perioperative events, including complications and conversions to thoracotomy. The trial was terminated early at 83 enrolled cases due to futility, with interim analysis indicating no likelihood of demonstrating superiority of RATS for the primary end-point. After exclusion of 6 patients, analysis revealed no statistically significant differences between the 2 arms in terms of perioperative complication rates, conversion rates, operative duration, or postoperative length of stay. However, RATS resulted in a significantly greater extent of lymphadenectomy, with higher median numbers of lymph node stations sampled (6 vs 4; *P* = .0002), hilar lymph nodes (7 vs 4; *P* = .0003), and mediastinal lymph nodes (7 vs 5; *P* = .0001). These findings indicate that while RATS did not improve perioperative outcomes compared to VATS, it may facilitate more extensive lymph node dissection.

Lastly, the RAVAL trial^6^ (NCT04762685) is an ongoing Canadian-led multicentre RCT aiming to randomize 600 patients to RATS or VATS lobectomy. In the early results of this trial, 164 patients with early-stage NSCLC were analysed following randomization to robotic-assisted lobectomy (RPL-4 (robotic portal lobectomy with 4 arms); *n* = 81) or VATS-lobectomy (*n* = 83). The primary aim was to evaluate cost-effectiveness and patient-reported health utility over a 12-month period. Baseline characteristics, including demographics, comorbidity burden, pulmonary function, and tumour profile, were comparable between groups. Health utility was assessed using the EQ-5D-5 l at multiple postoperative time points. At 12 weeks, the mean health utility score was significantly higher in the RPL-4 group compared to VATS (0.85 ± 0.10 vs 0.80 ± 0.19; *P* = .02). Additionally, the robotic approach was associated with a higher lymph node yield [median 10 (IQR 8-13) vs 8 (IQR 5-10); *P* = .003]. Using seemingly unrelated regression models adjusted for baseline utility and imputation for missing data, the incremental cost-effectiveness ratio (ICER) of RPL-4 compared to VATS-lobectomy was calculated at $14 925.62 per quality-adjusted life year (QALY) gained (95% CI: $6843.69-$23 007.56). These early data suggest that RPL-4 is a cost-effective alternative to VATS lobectomy within publicly funded health systems and may offer modest improvements in patient-reported health utility and oncological thoroughness, as reflected by increased lymph node sampling.

These studies represent the highest level of evidence to date comparing RATS to conventional techniques and support its safety, with potential benefits in lymph node dissection, conversion rates, and patient-centred outcomes. Meta-analytical data has further consolidated the benefit of RATS over VATS especially in terms of lymphadenectomy and control of intraoperative bleeding.^27^ The upcoming prospective RCT with McMaster University (RAVAL-4) is expected to provide further high-level evidence comparing RATS to VATS/open surgery.^9^  **Table 2** summarizes the key findings from the randomized data in robotic thoracic surgery.

**Table 2.** Key Findings From the Randomized Data in Robotic Thoracic Surgery

Trial	Sample/population	Primary outcomes	Key findings (RATS vs VATS)	Direction of effect
RVLob (NCT03134534)	*n* = 320; resectable NSCLC; single-centre, China	3-year overall survival (OS); DFS	OS: 94.6% vs 91.5% (non-inferior) DFS: 88.7% vs 85.4% (*P* = NS) LN yield: Higher with RATS (stations + nodes) Peri-op outcomes: similar Cost: higher with RATS	Oncological non-inferiority; improved nodal assessment
ROMAN	*n* = 71 analysed; early-stage NSCLC; multinational	Peri-operative complications; conversions	Complications: no difference Conversions: no difference LN dissection: RATS significantly more stations, N1 + mediastinal nodes Length of stay and operative time: Similar	Better lymphadenectomy with RATS; otherwise, equivalent
RAVAL	*n* = 164; early-stage NSCLC; Canada/US/France	Cost-effectiveness; HRQoL	QoL: 12-week EQ-5D slightly higher with RATS (0.85 vs 0.80) LN yield: higher with RATS (10 vs 8 nodes) ICER: ∼$14 926 per QALY (cost-effective) Complications: similar	Cost-effective RATS adoption; modest QoL and LN benefits

A further area of utility for the robotic platform is in the surgery of the mediastinum; with the improved ergonomics and wristed instrumentation, dissection and mobilization of structures in a restricted space is much easier. One of the earliest series reporting on robotic surgery in the mediastinum comes from mainland China^28^; 167 patients with a range of conditions from thymoma, thymic cyst, schwannoma, bronchogenic, and foregut cysts underwent resection over an 8-year period. The mean post-operative length of stay was 4.09 days with an overall complication rate of 3%. This supported the safety and feasibility of the platform in this patient population. Resection of thymoma both in and out of the setting of Myasthenia Gravis (MG), as well as non-thymomatous resection for MG, has been performed through minimal access routes for over a decade. Traditional teaching has stated that the gold standard for complete resection is via the transsternal route, but data have shown no difference in the completeness of resection whether an open or minimally invasive route is chosen.^29^^,^^30^ The robotic approach has certainly cemented the minimal access route as efficacious in this regard. Data from 15 Italian centres have demonstrated a 98.6% R0 resection rate in 669 patients with thymic epithelial malignancies who underwent robotic resection. Of this cohort, 312 had associated myasthenia gravis; there was no peri-operative mortality, and the overall complication rate was 7.7%. Complete thymectomy was performed in 98% of cases (*n* = 657).^31^ A large multicentre European Society of Thoracic Surgery (ESTS) database analysis compared robotic-assisted thymectomy and video-assisted thymectomy (VATS) for thymic epithelial malignancies, focusing on resection quality and long-term outcomes. Using data from 899 minimally invasive thymectomies performed between 2001 and 2021, the authors conducted a propensity-matched analysis of 732 patients (366 RATS; 366 VATS). After adjusting for tumour stage and relevant covariates, robotic surgery was associated with significantly lower odds of incomplete (R1) resection (OR 0.203, 95% CI 0.13-0.317; *P* < .001). Despite this difference in pathological radicality, overall survival and disease-free survival were equivalent between RATS and VATS. No survival advantage was observed for either technique within the follow-up period.^18^ Longer follow-up and data maturation may clarify whether improved R0 rates translate into survival differences, but confirmation would likely need to be sought in the setting of a randomized controlled trial.

## ADVANCES IN SINGLE-PORT ROBOTIC SURGERY

Recent studies have explored the feasibility and early outcomes of SP robotic platforms in thoracic surgery. The distinction being that there now exist dedicated single-port systems as opposed to the use of current modern-day multi-arm platforms (eg, Da Vinci Xi) used in uni-portal configuration, and the former is the focus of this section. In a prospective feasibility study, Zervos *et al.*^32^ evaluated SP robotic-assisted pulmonary lobectomy, reporting successful completion in the majority of cases consistent with minimally invasive surgery standards. In this 6-centre U.S. study, 19 patients (benign *n* = 1; malignant *n* = 18) underwent SP lobectomy without conversion to multiport, thoracoscopic, or open approaches. No intraoperative adverse events or unanticipated device effects were reported. Thirteen postoperative adverse events occurred, 4 of which were Clavien–Dindo grade III. Oncological quality was maintained, with a 100% R0 resection rate, a median of 6.5 (IQR 6-8) nodal stations sampled, and a median of 17.5 (IQR 7-34) lymph nodes resected per patient.

Complementing these findings, Lee *et al.*^33^ published their initial institutional experience of over 100 consecutive SP robotic thoracic procedures, demonstrating acceptable safety, low complication rates, and a clear learning curve plateau after approximately 20-25 cases. Median operative times and chest tube durations varied by procedure, with anatomical pulmonary resections averaging 187.2 ± 55.8 min and 2.5 ± 1.5 days, respectively. There were no conversions to thoracotomy or sternotomy; 1 patient required conversion to VATS and 2 required an additional port. Only 2 patients experienced complications exceeding Clavien–Dindo grade IIIa. Use of the multi-port Xi system through a uniportal (subxiphoid) approach for thymectomy has demonstrated efficacy,^34^ and further evolution of this has been to use the SP robotic system also from the subxiphoid approach in a similar patient cohort.^35^ This group from South Korea,^35^ directly compared SP (RATS) to SP (VATS) in the setting of thymectomy and demonstrated good feasibility of the robotic system with a lower conversion rate to multi-port surgery (0% vs 20%, *P *= .05), shorter chest tube drainage duration (1.32 ± 0.75 vs 2.00 ± 1.29 days, *P *= .003), and a shorter postoperative hospital stay (2.52 ± 1.00 vs 5.08 ± 5.20 days, *P *= .003). There are, however no direct comparisons in this setting between SP and multi-port robotic systems.

Taken together, these early series indicate that SP robotic thoracic surgery is technically feasible and can be performed safely in experienced hands, including for complex resections (post-induction treatment, bronchoplastic resections, high nodal disease burden, single segments, particularly in the basal territories). However, widespread adoption remains limited, with current experience concentrated in high-volume centres and, in some cases, under industry-led initiatives. Further independent, comparative studies are needed to determine its reproducibility, define patient selection, and clarify whether potential ergonomic or cosmetic advantages translate into measurable clinical benefits.

## TECHNICAL INNOVATIONS AND INTEGRATION

### Evolution of robotic platforms

Technological advancements are rapidly redefining the robotic thoracic surgery landscape. The current generation of robotic systems, particularly the da Vinci Xi, has already revolutionized thoracic surgery by offering 3D visualization, wristed instruments with 7 degrees of freedom, and stable camera platforms.^36^ However, the field is now poised for a new wave of disruptive innovations. The introduction of competing platforms such as Medtronic Hugo, CMR Versius, and the Intuitive dV5 has intensified technological development while promoting cost reduction and modularity.^37^ Both systems feature smaller footprints and independent arm configurations that can be adapted to varying operative fields, offering greater flexibility for multi-specialty theatres. This has been further compounded by the role of SP systems, as alluded to above.

### Intraoperative visualization: near infrared fluorescence

Integration of near-infrared fluorescence (NIRF) imaging for lymph node mapping and vascular delineation is now routine in many centres.^38^ In a prospective single-centre cohort (December 2022-April 2024),^39^ 79 patients with clinical stage I NSCLC ≤3 cm were planned for robotic segmentectomy using preoperative 3-dimensional (3D) lung reconstruction and, in most cases, NIRF with indocyanine green (ICG). Of 76 patients undergoing surgery, 3D reconstruction was successful in 88.16% (67/76), with ICG administered in 68.66% (46/67) and no dye-related complications. Segmentectomy was completed as planned in 80.60% (54/67), with conversion to lobectomy required in only 8.96% (6/67). Preoperative plans were altered after 3D reconstruction in 36.07% (22/61), significantly increasing surgeon confidence (*P* < .001). Thirty-day mortality was 1.49% (1/67). These results demonstrate that 3D reconstruction in robotic segmentectomy is feasible, safe, and associated with low conversion rates and improved operative planning.

### Pre-operative planning and navigation

Future developments are focusing on augmented reality (AR) overlays that incorporate preoperative 3D reconstructions, facilitating precise segmental resections and tumour localization. Sadeghi *et al.*^40^ present the first-in-human demonstration of an artificial intelligence (AI)–assisted AR platform integrated with RATS for anatomical lung resection. The system, developed in collaboration with MedicalVR, employs PulmoVR’s deep learning–based segmentation algorithm trained on 126 annotated CT datasets from lung resection patients to automatically generate high-fidelity 3D reconstructions of pulmonary arteries, veins, lobes, and airways. These reconstructions are transformed into interactive simulated realities (iSRs) using finite element method simulations, enabling deformable virtual 3D lung models that can be manipulated in real time to account for intraoperative deformation and compression. To address the challenge of visual obstruction by robotic instruments, the authors incorporated a real-time instrument de-occlusion algorithm, segmenting and digitally rendering non-organic items overlaid on the patient-specific 3D anatomy. This integration allows surgeons to maintain continuous visual access to key anatomical structures during RATS without loss of orientation. The technology was applied during a robotic lobectomy, demonstrating technical feasibility, safe integration into the operative workflow, and high visual fidelity of anatomical representation. Potential advantages include overcoming limitations of static preoperative imaging, improving precision in dissection, enhancing intraoperative navigation, and reducing cognitive load for the surgeon. The authors highlight current limitations, such as the inability to automatically deform virtual models based on real-time intraoperative changes and call for further validation in larger patient cohorts.

### AI-driven intraoperative analytics

AI is also beginning to play a role in intraoperative analytics and decision support. Prototype algorithms can detect critical structures, track instrument motion, and provide real-time feedback to reduce intraoperative errors.^41^ Fully autonomous robotic steps remain aspirational, but semi-autonomous suturing and precision cutting are already under active investigation.

Together, these developments illustrate a progressive but uneven technological evolution within robotic thoracic surgery, in which established tools such as NIRF imaging coexist with early-phase innovations including 3D reconstruction, AR overlays, and AI-enabled analytics. Although evidence is currently heterogeneous and often limited to feasibility studies, early data suggest that digital augmentation may enhance operative planning, improve anatomical precision, and support intraoperative decision-making. However, these technologies remain adjunctive, and their widespread integration will depend on multicentre validation, platform standardization, and demonstration of reproducible clinical benefit. Overall, thoracic robotics is transitioning from mechanical enhancement to a data-driven, image-guided, and decision-supported surgical ecosystem.

## TRAINING, CREDENTIALING, AND WORKFORCE DEVELOPMENT

The global expansion of robotic thoracic surgery has exposed significant gaps in training infrastructure, credentialing standards, and workforce preparedness. Traditional apprenticeship models, which are heavily reliant on open and VATS cases, are increasingly challenged by the growing robotic caseload. Studies suggest that the learning curve for robotic lobectomy can range between 15 and 30 cases, shorter than the learning curve for VATS due to the ergonomic advantages and intuitive controls of robotic systems.^1^^,^^42^ However, the steep costs associated with training (including simulator access and dedicated robotic time) limit opportunities for many trainees. High-fidelity simulation (virtual reality [VR] simulators, dry-lab and hybrid models) reliably improves operative skills and transfers to the operating theatre when embedded in a curriculum. Systematic reviews and meta-analyses demonstrate that VR simulator training shortens task completion times, reduces errors, and improves technical metrics that translate to clinical performance.^43^ Practical deployment can include commercially available platforms (eg, dV-Trainer, SimNow) and low-cost home models that provide skill maintenance; randomized data show that deliberate practice on home simulation models improves subsequent performance on validated robotic simulators.^43^^,^^44^ Modern robotic simulators (eg, Mimic dV-Trainer)^45^ allow trainees to acquire psychomotor skills before live cases, while virtual reality modules can simulate complex scenarios such as sleeve resections.^14^^,^^15^ Emerging AI-driven platforms are being developed to monitor trainee performance metrics (eg, instrument motion economy, force feedback), enabling objective competency-based assessment.

Tele-proctoring and remote proctorship have emerged as pragmatic solutions to bridge geographic and mentor availability gaps, enabling expert guidance for surgeons at centres with limited local expertise.^46^ Pilot studies demonstrate high trainee and proctor satisfaction, safe completion rates, and non-inferior procedural metrics compared with on-site proctoring, supporting wider adoption with robust technology and standardized protocols.^47^

In the UK, the SORTS UK (Survey of Robotic Training in Surgery) initiative is the first national effort to assess robotic exposure among cardiothoracic trainees. Early findings indicate that fewer than 50% of trainees have regular access to robotic platforms, and structured case logs are rarely maintained. The initiative highlights the need for national credentialing standards, integration of robotics into the Joint cardiothoracic curriculum, and the establishment of high-fidelity simulation training hubs. Modern curricula should align simulation drills to competency domains (camera control, suturing, dissection, energy control) and incorporate objective automated performance metrics (APMs) to quantify motion economy, efficiency, and error rates. Recent systematic reviews of robotic curricula identify core elements: didactic teaching, VR simulation, dry-lab tasks, bedside assisting, proctored cases, and formal assessment with milestone progression.^48^ While high-income countries are developing structured pathways for robotic training, access remains limited in low- and middle-income settings.^49^^,^^50^ Collaboration through international fellowships, proctorship programs, and virtual platforms may bridge this gap.

## ROBOTIC SURGERY IN THE ERA OF PRECISION MEDICINE

As surgical strategies evolve towards tissue preservation and nodal precision, robotic platforms are uniquely positioned to integrate molecular and imaging-guided decision-making. Advances in circulating tumour DNA (ctDNA) and minimal residual disease (MRD) monitoring have the potential to guide surgical decisions, including resection margins and adjuvant therapy strategies.^51^ Robotic systems with AR capabilities could integrate molecular imaging data intraoperatively, helping surgeons target sub-centimetre lesions or guide segmental resections with unprecedented accuracy. Future robotic platforms may integrate real-time navigation technologies, akin to those used in neurosurgery, to enhance surgical planning and execution.

The expansion of indications for immunotherapy use has seen its clear prominence in the neoadjuvant setting for resectable lung cancer. The implications of which incur a rise in the number of cases being done after induction treatment, which brings a host of challenges. Technically speaking, mediastinal fibrosis, fibrocalcified lymph nodes, perifissural adhesions, and vascular fragility all add extra layers of complexity to the surgery. There is also the added burden of post-operative complications associated with more complex surgeries.^52^ Robotic lung resection has been shown to be safe and effective in the setting of post-induction treatment for cancer.^53^^,^^54^ A retrospective study of stage II-IIIB (N2) NSCLC patients undergoing surgery after neoadjuvant therapy compared 118 RATS cases with 317 VATS cases. Before and after propensity score matching, RATS was associated with significantly shorter operative time (194 vs 223 min) and a lower conversion rate to thoracotomy, particularly in cases complicated by fibrosis or limited exposure. R0 resection rates and blood loss were similar between approaches. RATS also yielded a higher number of dissected N2 nodes after matching. Multivariable analyses confirmed that RATS independently predicted reduced operative time and fewer conversions. Overall, RATS appeared advantageous in the neoadjuvant setting, offering improved operative efficiency, lower conversion risk, and enhanced nodal evaluation without compromising oncological completeness.^55^

## HEALTH ECONOMICS, ACCESS, AND EQUITY

The widespread adoption of robotic thoracic surgery has been partly hindered by its perceived high cost. While upfront capital expenditure for robotic platforms remains significant, growing evidence suggests that the total cost of care (including shorter length of stay, reduced complications, and faster recovery) may offset initial investment.^6^ Higher utilization times will inevitably improve cost-effectiveness. The entry of alternative platforms such as CMR Versius and Medtronic Hugo may be expected to increase competition and drive prices down, potentially improving cost-effectiveness and accessibility.

Despite these advancements, significant global disparities remain. Robotic programs are concentrated in high-income countries, with limited access in low- and middle-income regions due to financial, infrastructural, and training barriers.^14^^,^^50^ Meta-analytical data evaluated the costs and cost-effectiveness of robotic-assisted surgery in South Korea across multiple surgical specialties.^56^ The authors synthesized data from comparative studies assessing robotic, laparoscopic, and open surgical approaches, focusing on total hospital costs, operative costs, length of stay, and cost–utility outcomes. Overall, robotic surgery was consistently more expensive than both laparoscopic and open surgery, with higher operative and equipment-related costs identified as the main drivers of increased expenditure. Length of stay was often reduced in robotic cohorts, particularly in urological and gynaecological procedures, but the magnitude of this reduction was insufficient to offset higher procedural costs. Only a small number of studies attempted formal cost-effectiveness analysis, and these demonstrated that robotic surgery did not meet conventional willingness-to-pay thresholds in the South Korean context. Evidence for improved patient-reported outcomes or reduced complications was inconsistent and generally insufficient to justify the additional cost. The authors conclude that, despite clinical feasibility and certain perioperative advantages, RAS is not cost-effective in South Korea under current pricing structures and utilization patterns. Innovative solutions, such as hub-and-spoke models, where regional robotic centres support surrounding hospitals, have been proposed to enhance access, particularly in the UK’s NHS setting.

## CHALLENGES, CONTROVERSIES, AND FUTURE RESEARCH

While RATS offers technical advantages, several controversies persist regarding its clinical superiority, cost, and training implications. Critics argue that robotic surgery risks “indication creep,” where the technology is applied to cases with little evidence of benefit over VATS or open approaches.^19^ The heavy influence of device manufacturers has also raised ethical questions about adoption strategies and marketing. Despite the existing data, the field lacks robust randomized evidence to demonstrate any oncological superiority of minimally invasive techniques over open surgery. The forthcoming RAVAL-4 trial (McMaster University) is expected to provide evidence on perioperative outcomes, cost-effectiveness, and oncological end-points.^9^ Lastly, there is an urgent need for large-scale, prospective registries to capture real-world data on robotic outcomes, learning curves, and cost metrics. Such registries will be critical in defining evidence-based benchmarks and guiding future policy. The next decade of robotic thoracic surgery will likely be defined by platform convergence, AI integration, and less invasive access strategies. Taken together, such an ecosystem has the potential to deliver more consistent, equitable, and outcomes-focused care as the field enters its next decade of evolution.

## Data Availability

No new data were generated or analysed in support of this research.
